# Supplementary material for: Dosing patterns and dose effects of sacubitril/valsartan: A claims-based retrospective cohort study
Source: PLoS One. 2025 Mar 25;20(3):e0320216. doi: 10.1371/journal.pone.0320216 (PMC11936244; doi:10.1371/journal.pone.0320216)

Supplemental Materials for:

Dosing patterns and dose effects of sacubitril/valsartan: a claims-based retrospective cohort study

Authors:
Jillian M. Rung, PhD, Tyson S. Barrett, PhD, Keith LeJeune, PhD, Shannon B. Richards, RN, MSN, Amresh Raina, MD, Lawrence Sinoway, MD

Published in *PLOS ONE*. Corresponding author contact information:
Jillian M. Rung, Highmark Health. Mailing Address: 120 Fifth Avenue Place, Pittsburgh, PA 15222. Office phone: (412) 544-2040. E-mail: jillian.rung@highmarkhealth.org

Contents

Supplemental Methods and Results 3
Additional Coding Details and Definitions 3
Modeling Details, Covariates, and Estimates 3
R Packages 4
Model Results with All Observations 5
Exploratory Analyses of Dose Titration Patterns 5
Exploratory Analyses of Time to First Admission 7
STROBE Statement—Checklist of items that should be included in reports of *cohort studies*. 8
Table S1. Conditions and procedures reviewed to characterize members' medical history and categorize admissions with corresponding codes used for identification. 11
Table S2. Generic and brand names used in developing code sets for each medication class. 15
Table S3. National drug codes used for identifying claims for fills of Entresto (sacubitril/valsartan). 17
Table S4. Model-estimated differences between dose groups presented as ratios, test statistics, and the significance thereof from models with all observations. 18
Table S5. Medical characteristics (conditions and procedures) during members' 6 months of baseline overall and by dose. 19
Table S6. Effect sizes (exponentiated coefficients; exp[Beta]), confidence intervals, and p-values for individual coefficients and global effects from generalized linear models predicting total costs of care during member's follow-up time 21
Table S7. Effect sizes (incidence rate ratios), confidence intervals, and *p*-values for individual coefficients and global effects from generalized linear models predicting all-cause admissions during member's follow-up time. 23
Table S8. Effect sizes (incidence rate ratios), confidence intervals, and *p*-values for individual coefficients and global effects from generalized linear models predicting heart failure admissions during member's follow-up time. 25
Table S9. Comparisons between dose groups from sensitivity analyses of cost and admissions. 27
Table S10. Descriptive statistics for unadjusted cost and utilization outcomes on a per-member per-month (PMPM) basis by final dose 28
Table S11. Comparisons of the number of cardiologist encounters across groups of members who evidenced different dosing trajectories of SAC/VAL 29
Table S12. Model-estimated differences between dose groups for time to first all-cause and heart failure admission presented as ratios, test statistics, and the significance thereof from Cox models 30
Table S13. Effect sizes (hazard ratios), confidence intervals, and *p*-values for individual coefficients from Cox proportional hazards models predicting time to first admission during member's follow-up. 31
Figure S1. Flow diagram of the selection process for the study sample. 33
Figure S2. Unadjusted average total allowed spend per member per month (PMPM) as a function of months since starting SAC/VAL with line of best fit and 95% confidence intervals. 34
Figure S3. Unadjusted average all-cause inpatient admissions per member per month (PMPM) as a function of months since starting SAC/VAL with line of best fit and 95% confidence intervals. 35
Figure S4. Unadjusted average heart failure admissions per member per month (PMPM) as a function of months since starting SAC/VAL with line of best fit and 95% confidence intervals. 36

Supplemental Methods and Results

Additional Coding Details and Definitions

In addition to conditions represented in the Charlson Comorbidity Index, claims were reviewed for diagnoses and procedures relevant to HF and cardiovascular disease broadly within members' baselines. These additional aspects of medical history are listed across rows of Tables S1 and S5.

Evidence of reduced ejection fraction was based on having at least one non-diagnostic claim with a heart failure diagnosis code commonly associated with ejection fraction values ≤ 50%, as outlined in the main text. Claims were considered non-diagnostic based on Milliman Health Cost Guideline (HCG) grouper codes, assigned by Milliman MedInsight software. Twelve different codes capturing outpatient and professional charges for diagnostic, pathology, and lab services were used to categorize claims as diagnostic at the encounter-level; claims with qualifying diagnoses *without* these HCG codes were considered evidence of heart failure with reduced (or mildly reduced) ejection fraction.

Inpatient claims were used to determine admissions, which had to meet several additional criteria to be considered a hospital admission. Specifically, inpatient claims that had a place of service other than skilled nursing facility, inpatient and outpatient rehabilitation clinic, and hospice, were considered indicative of a (hospital) admit. Heart failure admissions required a heart failure diagnosis code in the primary diagnosis position.

Evaluation and management visits with cardiologists were identified from outpatient and professional claims with qualifying procedure codes (see Table S1 below) for exploratory analyses (see Exploratory Analysis of Cardiologist Encounters, below). In addition to the aforementioned criteria, the claim could not be associated with an emergency room visit, and the physician specialty listed on the claim had to be associated with cardiac medicine (e.g., cardiology, interventional cardiology, advanced heart failure and transplant cardiology).

Modeling Details, Covariates, and Estimates

All generalized linear models used a log link and accounted for differences in duration of follow-up across members by including follow-up months as an offset. For cost models, robust standard errors were used to account for unequal variances across dose groups in associated significance tests. For all models, diagnostics were performed, which included standard checks by model type (e.g., test of dispersion), as well as residual diagnostics on both raw and/or simulated residuals. When feasible, influential observations were identified using Cook's Distances and removed from models (total costs and all-cause inpatient admission models); the magnitude of the

model-estimated outcomes better resembled that of the raw data without these observations, and significance of results did not substantively differ. High influence values in the model for heart failure admissions could not be removed because these accounted for the majority of non-zero observations (76%). Due to this, all results pertaining to costs and all-cause admissions exclude high influence observations unless otherwise stated. Sensitivity analyses involving costs and all-cause admissions use the data with high influence observations removed based on the full subset. All *p*-values for dose group comparisons were corrected using the Tukey method for three tests (low vs. medium, medium vs. high, low vs. high).

Coefficients for categorical variables, or select levels thereof, could not be reliably estimated in certain instances in count-based models; when this occurred, the covariate or relevant level was dropped from the model. Covariates were dropped as follows: Census Region was not included in count-based models of all-cause or heart failure admissions; member's first dose of SAC/VAL was not included in sensitivity analyses for all-cause admissions; and an indicator for having cancer in the 6 months preceding the start of SAC/VAL was not included in sensitivity analyses for heart failure admissions. Sensitivity analyses for all outcomes in the 18+ follow-up months subset did not include "2022" as a possible starting year for SAC/VAL because those initiating SAC/VAL at that time could not have the 18 months required since only data through March of 2023 were queried for analyses. Finally, due to the relatively large number of members for whom duration of HF could not be calculated, this variable was omitted from models because inclusion of it would necessitate exclusion of additional members.

Reported model estimates were generated based on the means of all predictors with the exception of categorical variables, which were weighted proportionally based on sample prevalence (e.g., diabetes with complications was estimated to increase costs by 27.1%, and this effect was down-weighted to 5.9% in dose group estimates, which reflects that 21.8% of members had this condition).

R Packages

The primary packages used for the analyses were gtsummary,[1] rstatix,[2] adheRenceRX,[3] ggplot2,[4] ggpubr,[5] emmeans,[6] MASS,[7] sandwich,[8,9] performance,[10] and DHARMa,[15] referenced below:

1. Sjoberg DD, Whiting K, Curry M, Lavery JA, Larmarange J. Reproducible summary tables with the gtsummary package. *R J.* 2021;13(1):570-580.
2. *Rstatix: Pipe-friendly framework for basic statistical tests*. Version R package 0.7.2. 2023. https://rpkgs.datanovia.com/rstatix/

3. *adheRenceRX: Assess medication adherence from pharmaceutical claims data.* Version R package 1.0.0. 2020. https://github.com/btbeal/adheRenceRX

4. Wickham H. *Ggplot2*. 2 ed. Use r! Springer Cham; 2016:XVI, 260.

5. *ggpubr: 'Ggplot2' based publication ready plots*. Version R package 0.6.0. 2023. https://rpkgs.datanovia.com/ggpubr/

6. *emmeans: Estimated marginal means, aka least-squares means*. Version 1.10.0. 2021. https://CRAN.R-project.org/package=emmeans

7. Venables WN, Ripley BD. *Modern applied statistics with s*. 4 ed. Stat comput. Springer; 2002:XII, 498

8. Zeileis A. Object-oriented computation of sandwich estimators. *J Stat Softw*. 08/15 2006;16(9):1 - 16. doi:10.18637/jss.v016.i09

9. Zeileis A, Köll S, Graham N. Various versatile variances: An object-oriented implementation of clustered covariances in r. *J Stat Softw*. 10/07 2020;95(1):1 - 36. doi:10.18637/jss.v095.i01

10. Lüdecke D, Ben-Shachar MS, Patil I, Waggoner P, Makowski D. Performance: An R package for assessment, comparison and testing of statistical models. *J Open Source Softw*. 2021;6(60)

11. *DHARMa - residual diagnostics for hierarchical models*. Version R package 0.4.6. 2022. http://florianhartig.github.io/DHARMa/

Model Results with All Observations

Dose group comparisons were the same in terms of statistical significance when including all observations for analyses of total costs and all-cause admissions, although the magnitude of the ratios differed slightly (see Table S4 below). Note that comparisons are not provided for heart failure admissions because the analysis results presented in the main text include all observations.

Exploratory Analyses of Dose Titration Patterns

Two potential explanations for varying dose patterns across members were evaluated in exploratory analyses. The first analysis sought to understand whether dose titration may have been precluded by select conditions within members' medical histories, or the occurrence thereof after initiating SAC/VAL. For this analysis, all diagnosis codes appearing on members' claims during the baseline and follow-up period were reviewed for indications of hypotension (ICD-10 CM codes I95x), hyperkalemia (E87.5), and acute kidney injury (N17.9). Then,

members were categorized based on changes (or lack thereof) in their SAC/VAL dosing during follow-up: consistent dose (*n* = 1,907; the same dose the entire duration filling it), increased (*n* = 916; ended on a higher dose than what the member started on), and down or returned to start (*n* = 154; decreased relative to the starting dose or returned to starting dose following attempted upward titration). The proportion of members experiencing each of the AEs were compared as a function of dose trajectory using Chi-squared tests, with the magnitude of effect quantified as Cramer's *V*. Tests were conducted both within the baseline and follow-up periods.

The prevalence of hypotension (approximately 10%) and hyperkalemia (approximately 4%) during baseline was relatively low within each dose trajectory group. Acute kidney injury was more common, but slightly less common among those with consistent dosing (15%) than those who eventually de-escalated doses or increased doses (20% each; *V* = .06). Given that prevalence was similar across groups or lower among those with consistent dosing, recent history of these conditions does not seem to explain the subsequent lack of titration in those who did not titrate. During follow up, there were significant differences across dose trajectories in experience of hypotension and acute kidney injury (*p*s <.001) but not hyperkalemia. Inspection of the proportions within groups revealed that those who titrated downward or returned to their starting dose experienced these events at approximately 2x the rate of those who remained on the same dose or increased their dose (see Table 4 in main text). This finding suggests that those who titrated downward likely did so due to the experience of adverse events; and additionally, that lack of titration does not appear to be due to poor tolerability of one's initial dose. However, it is important to note that the selected conditions may be difficult to detect from review of medical claims alone; as such, the absolute rates identified may not be accurate. While tolerability appears similar across those who increased doses compared to those who remained on the same dose, this does not rule out the possibility that this finding indicates prescribers were attempting to *avoid* potential worsening of these conditions among those who did not titrate. Additional data are needed to make more definitive conclusions.

For the second exploratory analysis, we sought to determine whether dose titration was associated with provider specialty. Members were categorized into one of four groups based on *both* their dose trajectory and dosage: consistent low dose (*n* = 1,490), downward titration or titration with return to starting dose (*n* = 154), consistent medium or high dose (*n* = 417), or upward titration (*n* = 916). Differences in the number of evaluation and management encounters with cardiologists during members' follow-up periods was evaluated across these

groups while controlling for members' index year and number of follow-up months in a generalized linear model (negative binomial error distribution).

Those with different dose trajectories and dose levels differed in how often they had visits with a cardiologist during follow-up. Specifically, those who were on the same dose consistently had significantly fewer visits (estimate of 2.82 and 2.45 visits for those consistently on low and medium/high doses) than those who titrated and ended on a higher dose (estimate = 3.12) or those who titrated and ended on the same or lower dose from one's starting dose (estimate = 3.57) over the average follow-up (14.3 months). All pairwise comparisons except those between the latter two groups were significant. Ratios for group comparisons, test statistics, and *p*-values, are shown in Table S11.

Exploratory Analyses of Time to First Admission

We evaluated whether dose group differences were consistent with an alternative outcome associated with admissions: time to first admission. For these analyses, the time to first all-cause and heart failure admission served as the outcome in Cox proportional hazards models with robust standard errors. Robust standard errors were used due to some covariates violating proportional hazards assumptions, though visual inspection of residual plots indicated the violations were typically minor and likely significant due to the large sample size. The same covariates included in generalized linear models testing differences in the number of admissions were used in Cox models, unless their inclusion precluded model convergence. Model results focused on dose group comparisons are provided in Table S12, with full model results and a listing of covariates included in Table S13.

STROBE Statement—Checklist of items that should be included in reports of *cohort studies*.

Note that page numbers below are based on those from the submitted manuscript.

	Item No	Recommendation	Page No
**Title and abstract**	1	(*a*) Indicate the study's design with a commonly used term in the title or the abstract	1
		(*b*) Provide in the abstract an informative and balanced summary of what was done and what was found	2
**Introduction**			
Background/rationale	2	Explain the scientific background and rationale for the investigation being reported	3
Objectives	3	State specific objectives, including any prespecified hypotheses	3-4
**Methods**			
Study design	4	Present key elements of study design early in the paper	4-5
Setting	5	Describe the setting, locations, and relevant dates, including periods of recruitment, exposure, follow-up, and data collection	4-5
Participants	6	(*a*) Give the eligibility criteria, and the sources and methods of selection of participants. Describe methods of follow-up	4-5
		(*b*) For matched studies, give matching criteria and number of exposed and unexposed	n/a
Variables	7	Clearly define all outcomes, exposures, predictors, potential confounders, and effect modifiers. Give diagnostic criteria, if applicable	4-6, supplemental methods and Tables S1-S3
Data sources/ measurement	8*	For each variable of interest, give sources of data and details of methods of assessment (measurement). Describe comparability of assessment methods if there is more than one group	4-5, supplemental materials
Bias	9	Describe any efforts to address potential sources of bias	4-5, 6-8, supplemental methods
Study size	10	Explain how the study size was arrived at	4-5, Figure S1
Quantitative variables	11	Explain how quantitative variables were handled in the analyses. If applicable, describe which groupings were chosen and why	6-7, supplemental methods

	Item No	Recommendation	Page No
Statistical methods	12	(*a*) Describe all statistical methods, including those used to control for confounding	(a) 7, supplemental methods
		(*b*) Describe any methods used to examine subgroups and interactions	(b) n/a
		(*c*) Explain how missing data were addressed	(c) 5, supplemental methods (regarding covariates)
		(*d*) If applicable, explain how loss to follow-up was addressed	(d) n/a
		(*e*) Describe any sensitivity analyses	(e) 7, supplemental methods
**Results**			
Participants	13*	(a) Report numbers of individuals at each stage of study—e.g. numbers potentially eligible, examined for eligibility, confirmed eligible, included in the study, completing follow-up, and analysed	(a) Figure S1
		(b) Give reasons for non-participation at each stage	(b) n/a
		(c) Consider use of a flow diagram	(c) Figure S1
Descriptive data	14*	(a) Give characteristics of study participants (e.g. demographic, clinical, social) and information on exposures and potential confounders	(a) 8, 9, 13, Tables 1 and 2, Table S5
		(b) Indicate number of participants with missing data for each variable of interest	(b) Figure S1, Table 1
		(c) Summarise follow-up time (e.g., average and total amount)	(c) Table 1
Outcome data	15*	Report numbers of outcome events or summary measures over time	13-15; Figure 2, Figures S2-S4
Main results	16	(a) Give unadjusted estimates and, if applicable, confounder-adjusted estimates and their precision (e.g., 95% confidence interval). Make clear which confounders were adjusted for and why they were included	(a) Unadjusted estimates in Table S10 and Figure S2-S4. Confounder documentation and explanation, pg. 6-8 and supplemental methods, Tables S6-S8. Confounder adjusted est., pg. 13-15, Figure 2, Tables S6-S8.
		(b) Report category boundaries when continuous variables were categorized	(b) n/a
		(c) If relevant, consider translating estimates of relative risk into absolute risk for a meaningful time period	(c) n/a
Other analyses	17	Report other analyses done—e.g. analyses of subgroups and interactions, and sensitivity analyses	15-18, Tables 4 and S6-S13

	Item No	Recommendation	Page No
**Discussion**			
Key results	18	Summarise key results with reference to study objectives	15
Limitations	19	Discuss limitations of the study, taking into account sources of potential bias or imprecision. Discuss both direction and magnitude of any potential bias	18-20
Interpretation	20	Give a cautious overall interpretation of results considering objectives, limitations, multiplicity of analyses, results from similar studies, and other relevant evidence	15-20
Generalisability	21	Discuss the generalisability (external validity) of the study results	18-20
**Other information**			
Funding	22	Give the source of funding and the role of the funders for the present study and, if applicable, for the original study on which the present article is based	n/a

Note: An Explanation and Elaboration article discusses each checklist item and gives methodological background and published examples of transparent reporting. The STROBE checklist is best used in conjunction with this article (freely available on the Web sites of PLoS Medicine at http://www.plosmedicine.org/, Annals of Internal Medicine at http://www.annals.org/, and Epidemiology at http://www.epidem.com/). Information on the STROBE Initiative is available at http://www.strobe-statement.org.

Table S1. Conditions and procedures reviewed to characterize members' medical history and categorize admissions with corresponding codes used for identification.

Condition/Procedure	Code Type(s)	Codes
Atrial fibrillation	ICD-10 CM	I480, I481, I4811, I4819, I482, I4820, I4821, I4891
Anxiety disorder	ICD-10 CM	F41xx, F4323xx, F4322xx
Coronary artery bypass graft	CPT and HCPCS	33511, 33512, 33513, 33514, 33516, 33517, 33518, 33519, 33521, 33522, 33523, 33533, 33534, 33535, 33536, 33510, 33508, 33509, 33510, 33530, 33572, 35500, 35600, S2205, S2206, S2207, S2208, S2209, 210083, 210088, 210089, 210344, 210444, 210483, 210488, 210489, 210493, 210498, 210499, 211083, 211088, 211089, 211098, 211099, 211344, 211444, 211483, 211488, 211489, 211493, 211498, 211499, 021008C, 021008F, 021008W, 021009C, 021009F, 02100AF, 02100J3, 02100J8, 02100J9, 02100JC, 02100JF, 02100JW, 02100K3, 02100K8, 02100KC, 02100KF, 02100ZF, 02103D4, 021048C, 021048F, 021048W, 021049C, 021049F, 021049W, 02104A3, 02104A8, 02104A9, 02104AC, 02104AF, 02104AW, 02104D4, 02104J3, 02104J8, 02104J9, 02104JC, 02104JF, 02104JW, 02104K3, 02104K8, 02104K9, 02104KC, 02104KF, 02104KW, 02104Z3, 02104Z8, 02104ZC, 02104ZF, 021108C, 021108F, 021109C, 021109F, 02110A8, 02110AC, 02110AF, 02110J3, 02110J8, 02110J9, 02110JC, 02110JF, 02110JW, 02110K8, 02110K9, 02110KC, 02110KF, 02110KW, 02110ZC, 02110ZF, 02113D4, 021148C, 021148F, 021148W, 021149C, 021149F, 02114A3, 02114A8, 02114A9, 02114AC, 02114AF, 02114AW, 02114D4, 02114J3, 02114J8, 02114J9, 02114JC, 02114JF, 02114JW, 02114K3, 02114K8, 02114K9, 02114KC, 02114KF, 02114KW, 02114Z8, 02114Z9, 02114ZC, 02114ZF, 210093, 210098, 210099, 211093, 212093, 212099, 213093, 021009W, 02100A3, 02100A8, 02100A9, 02100AC, 02100AW, 02100K9, 02100KW, 02100Z3, 02100Z8, 02100Z9, 02100ZC, 02104Z9, 021108W, 021109W, 02110A3, 02110A9, 02110AW, 02110K3, 02110Z3, 02110Z8, 02110Z9, 021149W, 02114Z3, 021209W, 02120Z3, 02120Z8, 02120Z9, 021309W, 02130AW, 02130Z9
Cardiorespiratory failure and shock	ICD-10 CM	R570, R092
Cardiac resynchronization therapy	CPT and HCPCS	33200, 33201, 33202, 33203, 33206, 33207, 33208, 33210, 33211, 33212, 33213, 33214, 33215, 33216, 33217, 33221, 33222, 33224, 33225, 33226, 33227, 33228, 33229, 33230, 33231, 33240, 33262, 33263, 33264, 71090, 93279, 93280, 93281, 93286, 93288, 93293, 93294, 93640, 93641, 93731, 93732, 93733, 93734, 93735, 93736, 93741, 93742, 93743, 93744, 02H43JZ, 02H44JZ, 02H60JZ, 02H63JZ, 02H64JZ, 02H73JZ, 02HK0JZ, 02HK3JZ, 02HK3NZ, 02HK4JZ, 02HL0JZ, 02HL3JZ, 02HL3MZ, 02HL4JZ, 02HN0JZ, 0387T, 0JH604Z, 0JH605Z, 0JH606Z, 0JH607Z, 0JH609Z, 0JH634Z, 0JH635Z, 0JH636Z, 0JH637Z, 0JH639Z, 0JH804Z, 0JH806Z, 0JH807Z, 0JH809Z, 0JH836Z, 0JH837Z, 0JH839Z, 4B02XSZ, C1068, C1069, C1071, C1105, C1118, C1125, C1126, C1127, C1128, C1129, C1130, C1131, C1132, C1133, C1134, C1135, C1136, C1143, C1144, C1149, C1153, C1156, C1157, C1162, C1180, C1181,

(continued)

Condition/Procedure	Code Type(s)	Codes
		C1182, C1183, C1184, C1311, C1315, C1316, C1317, C1318, C1354, C1355, C1358, C1359, C1779, C1785, C1786, C1898, C1899, C2619, C2620, C2621, C4000, C4001, C4002, C4003, C4004, C4005, C4006, C4007, C4008, C4009, C4300, C4301, C4302, C4303, C4304, C4305, C4306, C4307, C4308, C4309, C4310, C4311, C4312, C4313, C4314, C4315, C4316, C4317, C8501, C8518, C8519, C8520, C8750, E0610, E0615, G0298, G0448
Cardiac resynchronization therapy	ICD-10 CM	T82111A, T82111D, T82111S, T82121A, T82121D, T82121S, T82191A, T82191D, T82191S, Z45010, Z45018, Z950
Dyslipidemia	ICD-10 CM	E78xx
Edema and Fluid Overload	ICD-10 CM	R609, R601, R600, E8770, E8779, E877
Evaluation and Management Visits	CPT and HCPCS	99201-99205, 99211-99215, 99304-99310, 99315, 99316, 99318, 99324-99328, 99334-99337, 99339, 99340, 99341-99345, 99347-99350, 99374, 99375, 99377-99380, 99441-99443, 99457, 99495, 99496, G0071, G0402, G0438, G0439, G0466, G0467, G0468
Heart transplant	CPT and HCPCS	33927, 33928, 33929, 33935, 33945, 02RA0LZ, 02RA0MZ, 02YA0Z, 02YA0Z1, 02YA0Z2
Heart transplant	ICD-10 CM	Z941
Hypertension (essential)	ICD-10 CM	I10xx
Heart valve surgery	CPT and HCPCS	33361, 33362, 33363, 33364, 33365, 33366, 33367, 33368, 33369, 33405, 33406, 33410, 33411, 33412, 33413, 33430, 33465, 33475, 33496, 0256T, 0318T, 33440, 0483T, 0484T, 0646T, 02RF37H, 02RF37Z, 02RF3JH, 02RF3KH, 02RF47Z, 02RF48Z, 02RF4JZ, 02RG07Z, 02RG37H, 02RG37Z, 02RF07Z, 02RF08Z, 02RF0JZ, 02RF0KZ, 02RF38H, 02RF38Z, 02RF3JZ, 02RF3KZ, 02RF4KZ, 02RG08Z, 02RG0JZ, 02RG0KZ, 02RG38Z, 02RH07Z, 02RH08Z, 02RH0KZ, 02RJ08Z, X2RF032, 02RG38H, 02RG3JH, 02RG3JZ, 02RG3KH, 02RG3KZ, 02RG47Z, 02RG48Z, 02RG4JZ, 02RG4KZ, 02RH0JZ, 02RH37H, 02RH37Z, 02RH38H, 02RH38Z, 02RH3JH, 02RH3JZ, 02RH3KH, 02RH3KZ, 02RH47Z, 02RH48Z, 02RH4JZ, 02RH4KZ, 02RJ07Z, 02RJ0JZ, 02RJ0KZ, 02RJ37H, 02RJ37Z, 02RJ38H, 02RJ38Z, 02RJ3JH, 02RJ3JZ, 02RJ3KH, 02RJ3KZ, 02RJ47Z, 02RJ48Z, 02RJ4JZ, 02RJ4KZ
Implantable cardioverter defibrillator (Procedure Codes)	CPT and HCPCS	33223, 33230, 33231, 33240, 33249, 33262, 33263, 33264, 33270, 33271, 33273, 93282, 93283, 93284, 93287, 93289, 93295, 93640, 93641, 0319T, 0326T, 0320T, 0321T, 0322T, 0323T, 0324T, 0325T, 0327T, 0328T, 33224, 33225, 33226, 33241, 33243, 33244, 33245, 33246, 33272, 93642, 93644, 93260, 93261, C1721, C1722, C1777, C1882, C1895, C1896, C1899, G0448, 02H40JZ, 02H40KZ, 02H44KZ, 02H74KZ, 02HN4KZ, 0JH808Z, 0JH838Z, 02H43KZ, 02H60KZ, 02H63KZ, 02H64KZ, 02H70KZ, 02H73KZ, 02HK0KZ, 02HK3KZ, 02HK4KZ, 02HL0KZ, 02HL3KZ, 02HL4KZ, 02HN0KZ, 02HN3KZ, 0JH608Z, 0JH638Z, 4B02XTZ
Implantable cardioverter defibrillator	ICD-10 CM	Z95810, Z4502

(continued)

Ischemic heart disease	ICD-10 CM	I24xx
Extracorporeal membrane support	CPT and HCPCS	33946, 33947, 33948, 33949, 33951, 33952, 33953, 33954, 33955, 33956, 33958, 33959, 33962, 33963, 33964, 33965, 33966, 33984, 33985, 33986, 33987, 33988, 33989, 33957, 33969, 36822, 5A15223
Intra-aortic balloon device	CPT	33967, 33968, 33969, 33970, 33971, 33973, 33974, 93536
Left ventricular assist device	CPT and HCPCS	33975, 33976, 33977, 33978, 33979, 33980, 33981, 33982, 33983, 33990, 33991, 33992, 33993, 33995, 33997, 93750, Q0478, Q0479, Q0481, Q0484, Q0486, Q0491, Q0493, Q0495, Q0497, Q0498, Q0499, Q0501, Q0505, Q0506, Q0508, 02HA0QZ
Pulmonary edema	ICD-10 CM	J81, J810, J811
Peripheral artery disease	ICD-10 CM	I702, I70201, I70202, I70203, I70208, I70209, I7021, I70211, I70212, I70213, I70218, I70219, I7022, I70221, I70222, I70223, I70228, I70229, I7023, I70231, I70232, I70233, I70234, I70235, I70238, I70239, I7024, I70241, I70242, I70243, I70244, I70245, I70248, I70249, I7025, I7026, I70261, I70262, I70263, I70268, I70269, I7029, I70291, I70292, I70293, I70298, I70299, I703, I70301, I70302, I70303, I70308, I70309, I7031, I70311, I70312, I70313, I70318, I70319, I7032, I70321, I70322, I70323, I70328, I70329, I7033, I70331, I70332, I70333, I70334, I70335, I70338, I70339, I7034, I70341, I70342, I70343, I70344, I70345, I70348, I70349, I7035, I7036, I70361, I70362, I70363, I70368, I70369, I70369, I70391, I70392, I70393, I70398, I70399, I7040, I70401, I70402, I70403, I70408, I70409, I7041, I70411, I70412, I70413, I70418, I70419, I7042, I70421, I70422, I70423, I70428, I70429, I7043, I70431, I70432, I70433, I70434, I70435, I70438, I70439, I7044, I70441, I70442, I70443, I70444, I70445, I70448, I70449, I7045, I7046, I70461, I70462, I70463, I70468, I70469, I7049, I70491, I70492, I70493, I70498, I70499, I7050, I70501, I70502, I70503, I70508, I70509, I7051, I70511, I70512, I70513, I70518, I70519, I70521, I70522, I70523, I70528, I70529, I7053, I70531, I70532, I70533, I70534, I70535, I70538, I70539, I7054, I70541, I70542, I70543, I70544, I70545, I70548, I70549, I7055, I7056, I70561, I70562, I70563, I70568, I70569, I7059, I70591, I70592, I70593, I70598, I70599, I7060, I70601, I70602, I70603, I70608, I70609, I7061, I70611, I70612, I70613, I70618, I70619, I7062, I70621, I70622, I70623, I70628, I70629, I7063, I70631, I70632, I70633, I70634, I70635, I70638, I70639, I7064, I70641, I70642, I70643, I70645, I70648, I70649, I7065, I7066, I70661, I70662, I70663, I70668, I70669, I7069, I70691, I70692, I70693, I70698, I70699, I707, I70701, I70702, I70703, I70708, I70709, I70710, I70711, I70712, I70713, I70718, I70719, I7072, I70721, I70722, I70723, I70728, I70729, I7073, I70731, I70732, I70733, I70734, I70735, I70738, I70739, I7074, I70741, I70742, I70743, I70744, I70745, I70748, I70749, I7075, I7076, I70761, I70762, I70763, I70768, I70769, I7079, I70791, I70792, I70793, I70798, I70799, I708, 1709, I7091, I7092, I739, T82858A, T82856D, T82312A, T82322A, T82332A, T82392A, T82392D, T82392S, Z95820, Z9862, E0851, E0852, E0859, E0951, E0952, E0959, E1051, E1052, E1059, E115, E1151, E1152, E1159, E1351, E1352, E1359, I738, I7389, I798, I73
PCI (Procedure Codes)	CPT and HCPCS	92920, 92921, 92924, 92925, 92928, 92929, 92933, 92934, 92937, *(continued)*

		92938, 92941, 92943, 92944, 92973, 92980, 92981, 92982, 92984, 92995, 92996, 33572, 92920, 92921, 92924, 92925, 92928, 92929, 92933, 92934, 92937, 92938, 92941, 92943, 92944, 92973, 92980, 92981, 92982, 92984, 92984, 92995, 92996, 92998, C9600, C9601, C9602, C9603, C9604, C9605, C9606, C9607, C9608, G0290, G0291, 270346, 270356, 270446, 271346, 272346, 272376, 273346, 02700ZZ, 027034Z, 027035Z, 027036Z, 027037Z, 02703D6, 02703DZ, 02703EZ, 02703FZ, 02703GZ, 02703Z6, 02703ZZ, 027044Z, 02704DZ, 027134Z, 027135Z, 027136Z, 027137Z, 02713DZ, 02713EZ, 02713ZZ, 027234Z, 027236Z, 027237Z, 02723DZ, 02723GZ, 02723ZZ, 027334Z, 02733DZ, 02C00ZZ, 02C03Z6, 02C03ZZ, 02C13ZZ, 02C23ZZ, X2C0361
Percutaneous coronary intervention	ICD-10 CM	Z955, Z9861, V4582
Revascularization	CPT and HCPCS	37221, 37235, 92937, C1981, Z955, 37220, 37234, 92934, C1930, C9608, 37222, 0238T, 92938, C3551, Z9861, 37223, 0066, 92941, C7531, 37224, 3601, 92943, C7532, 37225, 3602, 92944, C7533, 37226, 3603, 92982, C7534, 37227, 3605, 92984, C7535, 37228, 37799, 92984, C9600, 37229, 3950, 92995, C9601, 37230, 92920, 92996, C9602, 37231, 92921, C1034, C9603, 37232, 92924, C1100, C9604, 37233, 92925, C1101, C9605, 92928, C1366, C9606, 92929, C1367, C9607, 92933, T82855A, T82855D, T82855S
Sleep apnea	ICD-10 CM	G477, G473, G4730, G4731, G4732, G4733, G4734, G4735, G4736, G4737, G4739
Shortness of breath	ICD-10 CM	R0602
Substance use disorder (including alcohol)	ICD-10 CM	F10, F101, F10180, F10280, F10980, F11188, F12, F12180, F12280, F129, F12980, F13280, F13980, F14, F14180, F14280, F14980, F15180, F15280, F15980, F16, F161, F16180, F162, F16980, F18980, F19, F191, F1914, F19159, F19180, F19188, F19280, F19980
Tachycardia	ICD-10 CM	I471, I47, I479, I472, R000, I4719, I4710, I4711, G90A
Heart failure	ICD-10 CM	I50xx, I0981, I97130, I97131, I110, I132
Reduced EF	ICD-10 CM	I5082, I5084, I502xx, I504xx

ICD-10CM = International Classification of Diseases, 10 revision, Clinical Modification. HCPCS = Healthcare procedure coding system. CPT = Current procedural terminology. EF = Ejection fraction.
Note: Codes were identified as exact matches unless the entry contains “xx”, which indicates that all codes beginning with the preceding letter and number combination were included. Due to the way codes are represented in the database used, no periods were included in ICD-10 CM codes.

Table S2. Generic and brand names used in developing code sets for each medication class.

Medication Class	Generic Name	Brand Names
ACE inhibitor	Benazepril	Lotensin<br>*lotrel
ACE inhibitor	Captopril	Capoten<br>*capozide<br>Vasotec
ACE inhibitor	Enalapril	Enalaprilat<br>Epaned<br>*lexxel<br>*vaseretic
ACE inhibitor	Fosinopril	-
ACE inhibitor	Lisinopril	Lytensopril<br>Prinivil<br>*prinzide<br>Qbrelis<br>Zestril<br>*zestoretic
ACE inhibitor	Moexipril	*uniretic<br>Univasc
ACE inhibitor	Perindopril	Aceon<br>*prestalia
ACE inhibitor	Quinapril	Accupril<br>*accuretic<br>*quinaretic
ACE inhibitor	Ramipril	Altace
ACE inhibitor	Trandolapril	Mavik<br>*tarka
ARB	Azilsartan	Edarbi<br>*edarbyclor
ARB	Candesartan	Atacand
ARB	Eprosartan	Teveten
ARB	Irbesartan	*avalide<br>Avapro
ARB	Losartan	Cozaar<br>*hyzaar
ARB	Olmesartan	Benicar<br>*tribenzor<br>*azor
ARB	Telmisartan	Micardis<br>*twynsta
ARB	Valsartan	Diovan<br>*exforge<br>*valturna<br>*byvalson
Beta blocker	Acebutolol	Sectral
Beta blocker	Atenolol	Tenormin<br>*tenoretic
Beta blocker	Betaxolol	Kerlone
Beta blocker	Bisoprolol	Zabeta<br>*ziac

Medication Class	Generic Name	Brand Names
		(continued)
Beta blocker	Carvedilol	Coreg
Beta blocker	Esmolol	Brevibloc
Beta blocker	Labetalol	Normodyne<br>Trandate
Beta blocker	Metoprolol	*dutroprol<br>Kapspargo<br>Lopressor<br>Toprol<br>Hypertensolol
Beta blocker	Nadolol	Corgard<br>*corzide
Beta blocker	Nebivolol	Bystolic<br>*byvalson
Beta blocker	Penbutolol	Levatol
Beta blocker	Pindolol	Visken
Beta blocker	Propranolol	Hemangeol<br>Inderal<br>*inderide<br>Innopran
SGLT2	Canagliflozin	Invokana<br>*invokamet
SGLT2	Dapagliflozin	Farxiga<br>*qtern<br>*xigduo
SGLT2	Empagliflozin	Jardiance<br>*glyxambi<br>*synjardy<br>*trijardy
SGLT2	Ertugliflozin	Steglatro<br>*segluromet<br>*steglujan

Note: Asterisk (*) indicates combination Medications. Hyphen (-) indicates not applicable.

Table S3. National drug codes used for identifying claims for fills of Entresto (sacubitril/valsartan).

NDC	Entresto Name/Dose
00078065920	ENTRESTO TAB 24-26 MG
00078-0659-20	ENTRESTO TAB 24-26 MG
00078-0659-35	ENTRESTO
00078065967	ENTRESTO TAB 24-26 MG
00078-0659-67	ENTRESTO TAB 24-26 MG
00078069620	ENTRESTO TAB 97-103 MG
00078-0696-20	ENTRESTO TAB 97-103 MG
00078-0696-35	ENTRESTO
00078069667	ENTRESTO TAB 97-103 MG
00078-0696-67	ENTRESTO TAB 97-103 MG
00078077720	ENTRESTO TAB 49-51 MG
00078-0777-20	ENTRESTO TAB 49-51 MG
00078077735	ENTRESTO
00078-0777-35	ENTRESTO
00078077767	ENTRESTO TAB 49-51 MG
00078-0777-67	ENTRESTO TAB 49-51 MG

NDC = National drug code.
Note. Dose information for analyses was obtained from a claim field specific to dose information, and not derived from name entries.

Table S4. Model-estimated differences between dose groups presented as ratios, test statistics, and the significance thereof from models with all observations.

Outcome	Dose Group Comparison	Est. Ratio	SE	df	Test Statistic (*t*/*z*)	*p*
Total Costs	Low / Medium	1.41	0.107	2932	4.491	<.001
	Low / High	1.68	0.154	2932	5.637	<.001
	Medium / High	1.19	0.087	2932	2.417	.042
All-Cause Admissions	Low / Medium	1.50	0.168	-	3.651	<.001
	Low / High	2.49	0.363	-	6.257	<.001
	Medium / High	1.66	0.214	-	3.904	<.001

Note. Entries of "-" for degrees of freedom appear when a *z*-statistic is used for the given comparison. Presented ratios are relative ratios (costs) and incidence rate ratios (admissions).

Table S5. Medical characteristics (conditions and procedures) during members' 6 months of baseline overall and by dose.

Measure Category/ Characteristic	Overall *N* = 2,977[1]	Final Dose Filled 24-26MG *n* = 1,605[1]	Final Dose Filled 49-51MG *n* = 809[1]	Final Dose Filled 97-103MG *n* = 563[1]	*p*-value[2]	Effect size[3]
**Charlson Comorbidity Index**						
CCI Score	3.00 (2.00, 4.00)	3.00 (2.00, 4.00)	3.00 (2.00, 4.00)	3.00 (2.00, 4.00)	.20	0.000
Cancer	270 (9.1%)	167 (10%)	64 (7.9%)	39 (6.9%)	.024	0.052
Cerebrovascular disease	466 (16%)	280 (17%)	111 (14%)	75 (13%)	.013	0.053
Congestive Heart Failure	2,656 (89%)	1,419 (88%)	728 (90%)	509 (90%)	.30	0.028
Chronic Pulmonary Disease	728 (24%)	387 (24%)	208 (26%)	133 (24%)	.60	0.018
Diabetes	516 (17%)	267 (17%)	144 (18%)	105 (19%)	.50	0.021
Diabetes with complications	647 (22%)	373 (23%)	157 (19%)	117 (21%)	.083	0.041
Hemiplegia	49 (1.6%)	27 (1.7%)	10 (1.2%)	12 (2.1%)	.40	0.024
Myocardial infarction	899 (30%)	512 (32%)	223 (28%)	164 (29%)	.078	0.042
Mild liver disease	197 (6.6%)	98 (6.1%)	60 (7.4%)	39 (6.9%)	.40	0.023
Peptic ulcer disease	40 (1.3%)	21 (1.3%)	10 (1.2%)	9 (1.6%)	.80	0.011
Peripheral vascular disease	1,066 (36%)	629 (39%)	269 (33%)	168 (30%)	<.001	0.080
Renal disease	670 (23%)	396 (25%)	155 (19%)	119 (21%)	.007	0.058
Rheumatic disease	101 (3.4%)	68 (4.2%)	20 (2.5%)	13 (2.3%)	.032	0.051
**Additional Conditions/Procedures**						
Total Recent CVD Procedures	0 (0, 1)	0 (0, 1)	0 (0, 1)	0 (0, 1)	<.001	0.005
Total Recent CVD Comorbidities	4 (3, 6)	4 (3, 6)	4 (3, 6)	4 (3, 6)	.005	0.003
Atrial fibrillation	1,157 (39%)	696 (43%)	282 (35%)	179 (32%)	<.001	0.102
Anxiety disorder	541 (18%)	308 (19%)	129 (16%)	104 (18%)	.15	0.036
Coronary artery bypass graft	77 (2.6%)	42 (2.6%)	20 (2.5%)	15 (2.7%)	>.99	0.005
Cardiorespiratory failure and shock	122 (4.1%)	74 (4.6%)	24 (3.0%)	24 (4.3%)	.15	0.035
Cardiac resynchronization therapy	406 (14%)	241 (15%)	97 (12%)	68 (12%)	.06	0.043
Dyslipidemia	2,177 (73%)	1,204 (75%)	587 (73%)	386 (69%)	.01	0.055
Edema and fluid overload	619 (21%)	346 (22%)	152 (19%)	121 (21%)	.20	0.030
Heart transplant	4 (0.1%)	1 (<0.1%)	0 (0%)	3 (0.5%)	.027	0.053
Hypertension (Essential)	2,316 (78%)	1,225 (76%)	639 (79%)	452 (80%)	.10	0.040
Heart valve surgery	34 (1.1%)	21 (1.3%)	9 (1.1%)	4 (0.7%)	.60	0.021

(continued)

Measure Category/ Characteristic	Overall N = 2,977[1]	Final Dose Filled 24-26MG n = 1,605[1]	49-51MG n = 809[1]	97-103MG n = 563[1]	*p*-value[2]	Effect size[3]
Implantable cardioverter defibrillator	573 (19%)	318 (20%)	143 (18%)	112 (20%)	.40	0.024
Ischemic heart disease	1,064 (36%)	609 (38%)	271 (33%)	184 (33%)	.016	0.050
Extracorporeal membrane support	8 (0.3%)	4 (0.2%)	1 (0.1%)	3 (0.5%)	.30	0.027
Intra-aortic balloon device	39 (1.3%)	26 (1.6%)	5 (0.6%)	8 (1.4%)	.10	0.038
Left ventricular assist device	27 (0.9%)	18 (1.1%)	3 (0.4%)	6 (1.1%)	.15	0.035
Pulmonary edema	382 (13%)	209 (13%)	101 (12%)	72 (13%)	>.99	0.007
Peripheral artery disease	538 (18%)	333 (21%)	128 (16%)	77 (14%)	<.001	0.077
Percutaneous coronary intervention	625 (21%)	372 (23%)	156 (19%)	97 (17%)	.003	0.060
Revascularization	333 (11%)	198 (12%)	91 (11%)	44 (7.8%)	.010	0.054
Sleep apnea	696 (23%)	327 (20%)	209 (26%)	160 (28%)	<.001	0.079
Shortness of breath	1,379 (46%)	737 (46%)	379 (47%)	263 (47%)	.90	0.009
Substance use disorder	0 (0%)	0 (0%)	0 (0%)	0 (0%)	-	-
Tachycardia	870 (29%)	458 (29%)	235 (29%)	177 (31%)	.40	0.024

[1]Median (IQR); n (%)
[2]Kruskal-Wallis rank sum test; Fisher's Exact test with simulated p-value (based on 2000 replicates).
[2]For Kruskal-Wallis tests, effect sizes are eta-squared; for Fisher’s Exact tests, effect sizes are Cramer’s V

Table S6. Effect sizes (exponentiated coefficients; exp[Beta]), confidence intervals, and p-values for individual coefficients and global effects from generalized linear models predicting total costs of care during member's follow-up time.

	All			3-8 Months			18+ Months		
**Characteristic**	**exp(Beta)**	**95% CI**	***p***	**exp(Beta)**	**95% CI**	***p***	**exp(Beta)**	**95% CI**	***p***
Dose			<.001			<.001			.009
Low	—	—		—	—		—	—	
Medium	0.82	0.75, 0.89	<.001	0.59	0.49, 0.73	<.001	0.89	0.79, 1.01	.075
High	0.68	0.60, 0.76	<.001	0.45	0.33, 0.61	<.001	0.79	0.69, 0.92	.002
First Dose			.725			.111			.827
Low	—	—		—	—		—	—	
Medium	1.01	0.93, 1.10	.786	1.27	1.03, 1.56	.023	1.00	0.89, 1.13	.947
High	1.08	0.91, 1.27	.398	1.24	0.89, 1.72	.207	1.07	0.85, 1.37	.557
Percent of Fills at Final Dose	0.73	0.61, 0.88	.001	0.49	0.30, 0.81	.001	0.88	0.68, 1.13	.259
PDC	1.62	1.01, 2.60	.053	1.95	0.84, 4.53	.155	0.97	0.46, 2.04	.929
Number of Dose Changes	1.06	1.02, 1.10	.011	1.07	0.96, 1.19	.222	1.07	1.01, 1.13	.013
Medicare Advantage	0.82	0.75, 0.89	<.001	0.81	0.69, 0.95	.007	0.83	0.71, 0.96	.003
Age at Index	1.00	1.00, 1.00	.418	1.00	1.00, 1.01	.560	0.99	0.99, 1.00	.010
Index During IP Admission [Yes]	1.01	0.92, 1.12	.794	1.00	0.84, 1.18	.956	1.07	0.91, 1.27	.384
Cardiac resynchronization therapy [Yes]	1.02	0.92, 1.14	.689	1.01	0.81, 1.26	.930	1.04	0.89, 1.21	.646
Percutaneous coronary intervention [Yes]	1.05	0.94, 1.18	.348	1.22	1.01, 1.48	.022	0.98	0.83, 1.16	.846
Revascularization [Yes]	1.24	1.05, 1.47	.016	0.99	0.73, 1.33	.937	1.14	0.86, 1.51	.376
Dyslipidemia [Yes]	0.98	0.90, 1.06	.572	0.95	0.82, 1.09	.444	0.99	0.88, 1.12	.908
Hypertension [Yes]	0.99	0.92, 1.08	.884	1.15	0.99, 1.34	.059	0.90	0.79, 1.02	.073
Sleep Apnea [Yes]	0.96	0.89, 1.04	.299	1.08	0.93, 1.27	.249	0.89	0.79, 1.01	.049
Peripheral Artery Disease [Yes]	0.90	0.82, 1.00	.033	0.88	0.73, 1.07	.121	1.01	0.87, 1.17	.917
Atrial Fibrillation [Yes]	1.03	0.96, 1.11	.378	1.08	0.95, 1.24	.207	1.03	0.93, 1.15	.559
Ischemic Heart Disease [Yes]	0.94	0.87, 1.02	.123	0.83	0.72, 0.96	.009	0.99	0.89, 1.10	.900
Myocardial Infarction [Yes]	0.90	0.84, 0.97	.009	0.92	0.81, 1.04	.209	0.91	0.81, 1.03	.118
Peripheral Vascular Disease [Yes]	1.10	1.01, 1.19	.017	1.05	0.90, 1.23	.474	1.09	0.96, 1.23	.159
Cerebrovascular Disease [Yes]	1.00	0.92, 1.09	.962	1.04	0.91, 1.20	.592	0.86	0.76, 0.98	.032
Rheumatic Disease [Yes]	1.12	0.97, 1.28	.180	1.17	0.93, 1.49	.272	1.08	0.83, 1.41	.542

(continued)

	All			3-8 Months			18+ Months		
**Characteristic**	**exp(Beta)**	**95% CI**	***p***	**exp(Beta)**	**95% CI**	***p***	**exp(Beta)**	**95% CI**	***p***
Diabetes with Complications [Yes]	1.31	1.20, 1.43	<.001	1.26	1.07, 1.49	.001	1.32	1.15, 1.52	<.001
Renal Disease [Yes]	0.96	0.89, 1.04	.308	0.98	0.85, 1.13	.807	0.94	0.84, 1.06	.327
Cancer [Yes]	1.20	1.08, 1.32	.001	1.10	0.91, 1.33	.310	1.15	1.00, 1.33	.085
Number of Additional Conditions	0.86	0.81, 0.91	<.001	0.87	0.77, 0.98	.019	0.88	0.80, 0.97	.019
Number of CVD Procedures	1.07	1.04, 1.11	<.001	1.09	1.03, 1.15	<.001	1.07	1.03, 1.11	.002
Index Year			.280			.011			.455
2020	—	—		—	—		—	—	
2021	1.03	0.96, 1.11	.422	1.17	0.96, 1.43	.113	1.03	0.94, 1.13	.466
2022	0.98	0.90, 1.05	.517	0.95	0.80, 1.13	.564			
Census Region			.750			.130			.935
Midwest	—	—		—	—		—	—	
Northeast	0.97	0.85, 1.11	.665	1.06	0.87, 1.30	.556	0.95	0.78, 1.16	.630
South	1.01	0.88, 1.17	.856	1.21	0.96, 1.52	.111	0.94	0.75, 1.18	.608
West	0.94	0.73, 1.21	.629	0.74	0.52, 1.05	.095	1.03	0.73, 1.45	.857
SVI Theme 3	0.93	0.81, 1.07	.295	0.73	0.56, 0.95	.014	1.13	0.91, 1.40	.264
Percentage without HS Diploma	1.00	1.00, 1.01	.444	1.01	1.00, 1.02	.104	1.0	0.99, 1.00	.193
Median Allowed PMPM (Baseline)	1.00	1.00, 1.00	<.001	1.00	1.00, 1.00	<.001	1.00	1.00, 1.00	<.001
Spend in the pre-index month (log)	1.10	1.08, 1.13	<.001	1.11	1.07, 1.16	<.001	1.08	1.05, 1.11	<.001
Baseline Use of ACEis	0.91	0.82, 1.02	.134	0.94	0.78, 1.15	.563	0.95	0.78, 1.15	.606
Baseline Use of ARBs	0.91	0.81, 1.03	.135	0.95	0.78, 1.16	.632	0.92	0.74, 1.13	.402
Baseline Use of Beta Blockers	0.93	0.83, 1.05	.258	0.94	0.76, 1.16	.573	0.97	0.79, 1.18	.742
Number of Baseline Therapies	1.01	0.92, 1.11	.819	0.93	0.80, 1.08	.372	1.07	0.90, 1.26	.459
Number of Baseline Observation Visits	1.03	0.85, 1.25	.702	1.11	0.80, 1.54	.389	0.80	0.57, 1.12	.162
Cardiologist Visit Before Index	1.02	0.96, 1.09	.477	0.96	0.86, 1.08	.559	1.07	0.96, 1.18	.195
Met Reduced EF Criteria	1.05	0.98, 1.12	.196	1.12	0.99, 1.26	.073	1.02	0.93, 1.13	.639
Number of Observations	2,901			1,034			961		

Note. Entries of “-” stand in place of the reference level for categorical variables that have more than two levels. Variables with “[Yes]” at the end of their name indicate two-level categorical variables; their reference level is no evidence of the given procedure or condition. The variable “Dose” (first-listed) is the dose group variable of primary interest in analyses for which pairwise comparisons are reported; this dosage reflects that of the final sacubitril/valsartan fill.
CI = Confidence Interval, CVD = Cardiovascular disease, EF = Ejection fraction, HS = high school, IP = Inpatient, PDC = Proportion of days covered, PMPM = Per-member per-month, SVI = Social Vulnerability Index.

Table S7. **Effect sizes (incidence rate ratios), confidence intervals, and *p*-values for individual coefficients and global effects from generalized linear models predicting all-cause admissions during member's follow-up time.**

	All			3-8 Months			18+ Months		
**Characteristic**	**IRR**	**95% CI**	***p*-value**	**IRR**	**95% CI**	**p-value**	**IRR**	**95% CI**	***p*-value**
Dose			<.001			<.001			<.001
Low	—	—		—	—		—	—	
Medium	0.66	0.54, 0.81	<.001	0.41	0.23, 0.70	.001	0.85	0.65, 1.10	.214
High	0.38	0.29, 0.49	<.001	0.26	0.11, 0.55	.001	0.44	0.31, 0.61	<.001
First Dose			.406						
Low	—	—							
Medium	1.12	0.89, 1.39	.316						
High	0.78	0.38, 1.43	.465						
Percent of Fills at Final Dose	0.52	0.35, 0.78	.002	0.39	0.10, 1.68	.205	0.66	0.39, 1.12	.121
PDC	0.22	0.07, 0.67	.008	0.30	0.03, 3.69	.341	0.18	0.04, 0.99	.048
Number of Dose Changes	1.11	1.03, 1.20	.011	1.02	0.71, 1.37	.923	1.16	1.04, 1.28	.006
Medicare Advantage	1.12	0.91, 1.39	.275	1.03	0.64, 1.67	.903	1.25	0.91, 1.71	.163
Age at Index	1.01	1.00, 1.02	.127	1.01	0.99, 1.03	.344	1.01	0.99, 1.02	.452
Index During IP Admission [Yes]	1.64	1.31, 2.03	<.001	1.65	1.03, 2.64	.039	1.53	1.10, 2.12	.012
Cardiac resynchronization therapy [Yes]	1.25	0.98, 1.59	.069	1.04	0.60, 1.80	.878	1.22	0.86, 1.73	.260
Percutaneous coronary intervention [Yes]	0.88	0.70, 1.10	.251	0.88	0.53, 1.43	.619	0.98	0.70, 1.35	.884
Revascularization [Yes]	1.88	1.31, 2.70	.001	0.82	0.32, 2.06	.677	1.61	0.95, 2.73	.079
Dyslipidemia [Yes]	0.94	0.77, 1.15	.535	0.95	0.61, 1.51	.831	0.97	0.73, 1.29	.828
Hypertension [Yes]	1.14	0.91, 1.43	.257	1.19	0.72, 2.06	.506	1.15	0.84, 1.59	.389
Sleep Apnea [Yes]	0.67	0.55, 0.81	<.001	0.68	0.43, 1.04	.076	0.71	0.53, 0.94	.017
Peripheral Artery Disease [Yes]	0.99	0.81, 1.21	.941	0.78	0.48, 1.26	.318	1.37	1.02, 1.84	.040
Atrial Fibrillation [Yes]	1.10	0.93, 1.31	.278	1.04	0.72, 1.50	.842	1.03	0.80, 1.34	.798
Ischemic Heart Disease [Yes]	0.85	0.70, 1.02	.081	0.65	0.43, 0.98	.039	0.82	0.62, 1.07	.141
Myocardial Infarction [Yes]	0.87	0.72, 1.05	.150	1.23	0.83, 1.80	.305	0.78	0.58, 1.03	.079
Peripheral Vascular Disease [Yes]	1.35	1.13, 1.63	.001	0.99	0.65, 1.50	.959	1.40	1.06, 1.85	.017
Cerebrovascular Disease [Yes]	1.05	0.86, 1.27	.630	1.06	0.70, 1.56	.776	0.85	0.62, 1.15	.301
Rheumatic Disease [Yes]	1.71	1.23, 2.32	.002	1.89	0.90, 3.54	.087	1.57	0.92, 2.53	.093
Diabetes with Complications [Yes]	1.63	1.35, 1.96	<.001	1.53	1.01, 2.31	.044	1.39	1.04, 1.84	.025
Renal Disease [Yes]	1.04	0.87, 1.25	.633	1.14	0.77, 1.68	.519	1.08	0.82, 1.42	.581
Cancer [Yes]	0.93	0.72, 1.17	.528	0.66	0.33, 1.19	.180	0.97	0.66, 1.37	.847
Number of Additional Conditions	0.72	0.62, 0.83	<.001	0.82	0.56, 1.18	.296	0.79	0.63, 0.98	.032
Number of CVD Procedures	1.17	1.10, 1.25	<.001	1.24	1.08, 1.42	.003	1.10	0.99, 1.22	.068

(continued)

Characteristic	All			3-8 Months			18+ Months		
	**IRR**	**95% CI**	***p*-value**	**IRR**	**95% CI**	**p-value**	**IRR**	**95% CI**	***p*-value**
Index Year			.002			.006			<.001
2020	—	—		—	—		—	—	
2021	0.75	0.63, 0.89	.001	0.77	0.46, 1.28	.306	0.65	0.52, 0.82	<.001
2022	0.98	0.81, 1.18	.825	0.51	0.33, 0.79	.002			
SVI Theme 3	0.92	0.65, 1.28	.610	0.49	0.21, 1.09	.081	0.88	0.54, 1.44	.624
Percentage without HS Diploma	1.00	0.99, 1.02	.706	1.01	0.98, 1.03	.683	1.00	0.98, 1.02	.684
Median Allowed PMPM (Baseline)	1.00	1.00, 1.00	.844	1.00	1.00, 1.00	.639	1.00	1.00, 1.00	.711
Spend in the pre-index month (log)	1.04	0.99, 1.09	.172	1.06	0.95, 1.20	.298	1.04	0.98, 1.12	.218
Baseline Use of ACEis	1.20	0.87, 1.69	.270	1.66	0.81, 3.65	.173	1.00	0.61, 1.68	1.00
Baseline Use of ARBs	1.24	0.89, 1.76	.209	2.11	1.02, 4.67	.043	1.06	0.63, 1.81	.837
Baseline Use of Beta Blockers	1.28	0.91, 1.81	.158	2.51	1.16, 5.82	.018	0.92	0.55, 1.58	.755
Number of Baseline Therapies	0.80	0.60, 1.05	.106	0.46	0.23, 0.84	.010	1.07	0.67, 1.63	.772
Number of Baseline Observation Visits	0.57	0.30, 0.96	.034	0.51	0.13, 1.35	.203	0.65	0.28, 1.30	.239
Cardiologist Visit Before Index	1.09	0.92, 1.28	.320	1.11	0.76, 1.59	.584	1.14	0.90, 1.45	.280
Met Reduced EF Criteria	1.01	0.84, 1.22	.878	0.96	0.64, 1.43	.821	0.98	0.75, 1.28	.875
Number of All-Cause Admits During Baseline	1.06	0.94, 1.20	.332	0.90	0.69, 1.18	.461	1.24	1.04, 1.47	.019
Number of Observations	2,841			1,040			920		

Note. Entries of "-" stand in place of the reference level for categorical variables that have more than two levels. Variables with "[Yes]" at the end of their name indicate two-level categorical variables; their reference level is no evidence of the given procedure or condition. Blank cells spanning all columns for an outcome indicate that a given variable or level thereof was dropped from the model due to an inability to reliably estimate its coefficient. The variable "Dose" (first-listed) is the dose group variable of primary interest in analyses for which pairwise comparisons are reported; this dosage reflects that of the final sacubitril/valsartan fill.

CI = Confidence Interval, CVD = Cardiovascular disease, EF = Ejection fraction, HS = high school, IRR = Incidence Rate Ratio, PDC = Proportion of days covered, PMPM = Per-member per-month, SVI = Social Vulnerability Index.

Table S8. Effect sizes (incidence rate ratios), confidence intervals, and *p*-values for individual coefficients and global effects from generalized linear models predicting heart failure admissions during member's follow-up time.

	All			3-8 Months			18+ Months		
**Characteristic**	**IRR**	**95% CI**	***p*-value**	**IRR**	**95% CI**	***p*-value**	**IRR**	**95% CI**	***p*-value**
Dose			.261			.531			.405
Low	—	—		—	—		—	—	
Medium	0.81	0.49, 1.34	.397	0.97	0.34, 2.80	.955	1.30	0.67, 2.49	.439
High	0.59	0.31, 1.11	.098	0.48	0.10, 2.18	.331	0.79	0.36, 1.69	.541
Dose_First			.829			.857			.850
24-26 mg	—	—		—	—		—	—	
49-51 mg	1.09	0.64, 1.84	.736	0.79	0.24, 2.37	.680	0.99	0.49, 1.90	.974
97-103 mg	1.38	0.43, 3.91	.544	1.27	0.15, 6.35	.794	1.58	0.24, 5.80	.551
Percent of Fills at Final Dose	0.42	0.16, 1.10	.076	2.18	0.20, 27.6	.527	0.31	0.11, 0.93	.037
PDC	0.58	0.04, 10.2	.703	0.49	0.01, 25.3	.709	0.20	0.00, 12.8	.437
Number of Dose Changes	1.04	0.84, 1.28	.713	1.23	0.68, 1.85	.442	1.21	0.96, 1.50	.103
Medicare Advantage	1.30	0.77, 2.19	.324	1.54	0.75, 3.21	.238	0.97	0.47, 2.07	.943
Age at Index	1.02	1.00, 1.04	.053	0.99	0.96, 1.02	.606	1.05	1.02, 1.08	.003
Index During IP Admission [Yes]	2.74	1.62, 4.66	.000	2.06	0.95, 4.36	.065	1.78	0.77, 3.99	.174
Cardiac resynchronization therapy [Yes]	1.34	0.74, 2.40	.326	1.08	0.46, 2.47	.858	1.15	0.52, 2.51	.733
Percutaneous coronary intervention [Yes]	1.84	1.09, 3.05	.022	2.47	1.33, 4.51	.005	1.29	0.62, 2.60	.490
Revascularization [Yes]	1.31	0.54, 3.16	.553	0.57	0.13, 2.11	.410	0.54	0.14, 1.93	.353
Dyslipidemia [Yes]	1.03	0.63, 1.73	.894	1.69	0.77, 4.16	.202	0.70	0.36, 1.40	.303
Hypertension [Yes]	0.90	0.53, 1.57	.718	0.93	0.43, 2.17	.865	0.59	0.28, 1.26	.167
Sleep Apnea [Yes]	1.03	0.66, 1.59	.901	1.35	0.74, 2.42	.331	0.63	0.31, 1.24	.186
Peripheral Artery Disease [Yes]	1.03	0.64, 1.65	.897	0.75	0.38, 1.48	.412	2.34	1.18, 4.62	.015
Atrial Fibrillation [Yes]	1.07	0.71, 1.61	.740	1.19	0.68, 2.12	.548	0.97	0.52, 1.82	.933
Ischemic Heart Disease [Yes]	0.70	0.44, 1.11	.135	0.58	0.30, 1.09	.092	0.92	0.47, 1.76	.807
Myocardial Infarction [Yes]	1.05	0.68, 1.60	.841	1.09	0.60, 1.93	.772	1.04	0.53, 1.98	.901
Peripheral Vascular Disease [Yes]	1.61	1.03, 2.51	.038	2.26	1.19, 4.28	.012	0.64	0.32, 1.25	.195
Cerebrovascular Disease [Yes]	0.71	0.43, 1.14	.158	0.74	0.37, 1.39	.358	0.51	0.21, 1.10	.089

(continued)

Characteristic	All IRR	All 95% CI	All *p*-value	3-8 Months IRR	3-8 Months 95% CI	3-8 Months *p*-value	18+ Months IRR	18+ Months 95% CI	18+ Months *p*-value
Rheumatic Disease [Yes]	2.18	0.97, 4.64	.057	2.48	0.83, 5.96	.096	4.73	1.57, 12.6	.008
Diabetes with Complications [Yes]	1.58	1.03, 2.43	.038	1.67	0.93, 2.98	.084	1.84	0.96, 3.48	.067
Renal Disease [Yes]	1.61	1.07, 2.42	.022	1.96	1.10, 3.50	.023	1.13	0.60, 2.06	.697
Cancer [Yes]	0.67	0.35, 1.21	.185						
Number of Additional Conditions	0.72	0.50, 1.03	.071	0.57	0.31, 1.01	.055	1.06	0.64, 1.71	.811
Number of CVD Procedures	1.16	0.99, 1.35	.060	1.13	0.92, 1.39	.233	1.19	0.94, 1.51	.147
Index Year			.328			.045			.331
2020	—	—		—	—		—	—	
2021	0.96	0.61, 1.50	.849	1.62	0.73, 3.87	.251	0.77	0.44, 1.31	.334
2022	1.31	0.82, 2.07	.235	0.77	0.37, 1.77	.510			
SVI Theme 3	1.53	0.68, 3.39	.299	0.47	0.14, 1.46	.196	1.28	0.38, 4.03	.676
Percentage without HS Diploma	1.01	0.98, 1.05	.405	1.04	0.99, 1.08	.114	0.99	0.93, 1.03	.593
Median Allowed PMPM (Baseline)	1.00	1.00, 1.00	.654	1.00	1.00, 1.00	.735	1.00	1.00, 1.00	.974
Spend in the pre-index month (log)	0.94	0.84, 1.06	.296	0.82	0.70, 0.97	.019	1.10	0.94, 1.31	.261
Baseline Use of ACEis	0.61	0.30, 1.23	.166	0.47	0.20, 1.15	.097	1.14	0.39, 3.67	.818
Baseline Use of ARBs	0.68	0.33, 1.42	.298	0.74	0.31, 1.86	.522	0.64	0.21, 2.07	.443
Baseline Use of Beta Blockers	1.00	0.48, 2.12	.998	0.63	0.24, 1.71	.359	2.38	0.67, 10.1	.189
Number of Baseline Therapies	1.38	0.78, 2.37	.264	1.65	0.83, 3.14	.150	1.53	0.56, 3.63	.386
Number of Baseline Observation Encounters	0.44	0.09, 1.51	.216	1.72	0.29, 4.75	.467	0.16	0.01, 1.05	.057
Cardio_Before_Index	1.02	0.68, 1.51	.923	1.45	0.83, 2.47	.187	0.69	0.36, 1.26	.227
Cardiologist Visit Before Index	1.82	1.14, 2.99	.012	2.01	1.05, 4.09	.035	1.59	0.79, 3.42	.201
Met Reduced EF Criteria	1.32	0.94, 1.85	.110	0.89	0.54, 1.40	.623	1.19	0.69, 1.93	.513
Number of Observations	2,977			1,087			966		

Note. Entries of "-" stand in place of the reference level for categorical variables that have more than two levels. Variables with "[Yes]" at the end of their name indicate two-level categorical variables; their reference level is no evidence of the given procedure or condition. Blank cells spanning all columns for an outcome indicate that a given variable or level thereof was dropped from the model due to an inability to reliably estimate its coefficient. The variable "Dose" (first-listed) is the dose group variable of primary interest in analyses for which pairwise comparisons are reported; this dosage reflects that of the final sacubitril/valsartan fill.

CVD = Cardiovascular disease, EF = Ejection fraction, HS = high school, IP = Inpatient, PDC = Proportion of days covered, PMPM = Per-member per-month, SVI = Social Vulnerability Index, IRR = Incidence Rate Ratio, CI = Confidence Interval.

Table S9. Comparisons between dose groups from sensitivity analyses of cost and admissions.

Modeled Outcome	Follow-Up Months Subset	Dose Group Comparison	Est. Ratio	SE	df	Test Statistic (*t*/*z*)	*p*
Total Costs	3 to 8	Low / Medium	1.69	0.175	988	5.09	< .001
	18 or more	Low / Medium	1.12	0.071	917	1.78	.177
	3 to 8	Low / High	2.32	0.367	988	5.34	< .001
	18 or more	Low / High	1.26	0.094	917	3.10	.006
	3 to 8	Medium / High	1.37	0.151	988	2.89	.011
	18 or more	Medium / High	1.13	0.073	917	1.85	.154
All-Cause Admissions	3 to 8	Low / Medium	2.40	0.67	-	3.14	.005
	18 or more	Low / Medium	1.17	0.15	-	1.17	.468
	3 to 8	Low / High	4.25	1.83	-	3.36	.002
	18 or more	Low / High	2.30	0.39	-	4.95	< .001
	3 to 8	Medium / High	1.77	0.75	-	1.34	.374
	18 or more	Medium / High	1.97	0.34	-	3.94	< .001
Heart Failure Admissions	3 to 8	Low / Medium	1.18	0.63	-	0.31	.948
	18 or more	Low / Medium	0.77	0.26	-	-0.77	.719
	3 to 8	Low / High	2.54	1.91	-	1.25	.426
	18 or more	Low / High	1.27	0.50	-	0.61	.814
	3 to 8	Medium / High	2.16	1.46	-	1.13	.494
	18 or more	Medium / High	1.65	0.62	-	1.32	.384

Note. *All p*-values are corrected using Tukey’s method for a family of three comparisons.

Table S10. Descriptive statistics for unadjusted cost and utilization outcomes on a per-member per-month (PMPM) basis by final dose.

	Final dose group		
**Duration of SAC/VAL**<br>Characteristic	**SAC/VAL, 24-26MG**	**SAC/VAL, 49-51 MG**	**SAC/VAL, 97-103 MG**
**3-8 Months**			
N	689	268	130
Mean Allowed PMPM	5,252 (10,136)	4,400 (7,013)	4,289 (9,722)
Median Allowed PMPM	1,688 (1,000, 3,056)	1,546 (929, 2,680)	1,523 (802, 2,308)
Mean AC Admits PMPM	0.06 (0.15)	0.03 (0.10)	0.02 (0.06)
Mean HF Admits PMPM	0.06 (0.15)	0.03 (0.10)	0.02 (0.06)
**9-17 Months**			
N	488	262	174
Mean Allowed PMPM	3,975 (4,417)	3,855 (4,010)	4,116 (7,467)
Median Allowed PMPM	1,511 (895, 2,288)	1,543 (918, 2,379)	1,734 (1,110, 2,323)
Mean AC Admits PMPM	0.03 (0.07)	0.03 (0.08)	0.02 (0.06)
Mean HF Admits PMPM	0.03 (0.07)	0.03 (0.08)	0.02 (0.06)
**18 + Months**			
N	428	279	259
Mean Allowed PMPM	3,226 (2,865)	2,868 (2,320)	2,856 (2,356)
Median Allowed PMPM	1,438 (841, 2,032)	1,229 (798, 1,791)	1,358 (792, 1,852)
Mean AC Admits PMPM	0.023 (0.042)	0.019 (0.032)	0.015 (0.032)
Mean HF Admits PMPM	0.023 (0.042)	0.019 (0.032)	0.015 (0.032)

Note: Single numbers in parentheses are SD and comma-separated numbers are first and third quartiles.

Table S11. Comparisons of the number of cardiologist encounters across groups of members who evidenced different dosing trajectories of SAC/VAL.

Dose Trajectory Comparison	Ratio	SE	*z*	*p*
All Low / Titrated to Starting or Lower	0.79	0.06	-3.31	.005
All Low / All Medium or High	1.15	0.06	2.58	.048
All Low / Titrated Upward	0.90	0.03	-2.65	.040
Titrated to Starting or Lower / All Medium or High	1.46	0.12	4.56	<.001
Titrated to Starting or Lower / Titrated Upward	1.15	0.08	1.88	.237
All Medium or High / Titrated Upward	0.79	0.04	-4.30	<.001

Note. “All Low” refers to all fills being on the lowest SAC/VAL dose, “All Medium or High” to having all fills on either the medium or highest SAC/VAL dose, “Titrated Upward” to having a dosage at one’s final fill that was higher than one’s starting, and “Titrated to Starting or Lower” to those who titrated downward relative to their starting dose or having filled doses other than one’s initial dose but had a final dose that was the same as one’s starting. Estimated means are reported in the body of the paper.

Table S12. Model-estimated differences between dose groups for time to first all-cause and heart failure admission presented as ratios, test statistics, and the significance thereof from Cox models.

Outcome	Dose Group Comparison	Est. Ratio	SE	df	Test Statistic (*t*/*z*)	*p*
Time to All-Cause Admission	Low / Medium	1.37	0.149	-	2.86	.012
	Low / High	2.27	0.312	-	5.95	< .001
	Medium / High	1.66	0.208	-	4.04	< .001
Time to Heart Failure Admission	Low / Medium	1.53	0.363	-	1.78	.158
	Low / High	1.76	0.526	-	1.91	.137
	Medium / High	1.16	0.309	-	0.54	.851

Note. Entries of "-" for degrees of freedom indicate a *z*-statistic is used for the given comparison. Presented ratios are hazard ratios.

Table S13. Effect sizes (hazard ratios), confidence intervals, and *p*-values for individual coefficients from Cox proportional hazards models predicting time to first admission during member's follow-up.

	All-Cause Admission			Heart Failure Admission		
**Characteristic**	**HR**	**95% CI**	***p*-value**	**HR**	**95% CI**	***p*-value**
Dose						
Low	—	—		—	—	
Medium	0.73	0.59, 0.91	.004	0.65	0.41, 1.04	.074
High	0.44	0.34, 0.58	.000	0.57	0.32, 1.02	.057
First Dose						
Low	—	—		—	—	
Medium	1.14	0.90, 1.44	.278	1.11	0.67, 1.85	.679
High	1.22	0.79, 1.89	.364	1.73	0.73, 4.10	.214
Percent of Fills at Final Dose	0.59	0.40, 0.87	.008	0.38	0.19, 0.78	.008
PDC	0.21	0.07, 0.64	.006	1.31	0.10, 17.6	.840
Number of Dose Changes	1.11	1.02, 1.21	.011	1.11	0.95, 1.30	.196
Medicare Advantage	1.04	0.84, 1.28	.726	1.19	0.79, 1.79	.397
Age at Index	1.01	1.00, 1.02	.063	1.02	1.00, 1.04	.070
Index During IP Admission [Yes]	1.62	1.28, 2.06	.000	2.46	1.57, 3.84	.000
Cardiac resynchronization therapy [Yes]	1.06	0.82, 1.36	.675	1.35	0.82, 2.21	.241
Percutaneous coronary intervention [Yes]	0.91	0.71, 1.16	.439	1.39	0.89, 2.16	.148
Revascularization [Yes]	1.40	0.92, 2.14	.118	1.15	0.51, 2.58	.738
Dyslipidemia [Yes]	0.88	0.72, 1.07	.199	0.95	0.60, 1.51	.824
Hypertension [Yes]	1.04	0.83, 1.29	.749	0.98	0.61, 1.58	.938
Sleep Apnea [Yes]	0.73	0.60, 0.89	.002	0.91	0.61, 1.37	.649
Peripheral Artery Disease [Yes]	1.02	0.82, 1.27	.860	1.06	0.67, 1.67	.804
Atrial Fibrillation [Yes]	1.04	0.87, 1.25	.675	1.01	0.68, 1.51	.966
Ischemic Heart Disease [Yes]	0.82	0.68, 0.99	.040	0.78	0.52, 1.16	.216
Myocardial Infarction [Yes]	0.93	0.77, 1.11	.416	1.07	0.75, 1.53	.694
Peripheral Vascular Disease [Yes]	1.20	1.00, 1.45	.056	1.41	0.96, 2.07	.083
Cerebrovascular Disease [Yes]	1.10	0.91, 1.34	.320	0.79	0.52, 1.19	.261

(continued)

Characteristic	All-Cause Admission HR	95% CI	*p*-value	Heart Failure Admission HR	95% CI	*p*-value
Rheumatic Disease [Yes]	1.32	0.91, 1.91	.145	2.07	1.08, 3.95	.028
Diabetes with Complications [Yes]	1.43	1.17, 1.74	.000	1.57	1.04, 2.36	.031
Renal Disease [Yes]	1.09	0.90, 1.30	.374	1.52	1.05, 2.20	.025
Cancer [Yes]	1.06	0.84, 1.35	.621	0.64	0.35, 1.17	.147
Number of Additional Conditions	0.84	0.72, 1.00	.046	0.79	0.57, 1.09	.151
Number of CVD Procedures	1.16	1.09, 1.24	.000	1.18	1.02, 1.36	.027
Index Year	—	—		—	—	
2020	—	—		—	—	
2021	0.79	0.66, 0.94	.010	0.86	0.58, 1.28	.454
2022	0.83	0.68, 1.02	.073	1.01	0.67, 1.53	.947
SVI Theme 3	1.00	0.71, 1.40	.993	1.47	0.73, 2.98	.285
Percentage without HS Diploma	1.01	0.99, 1.02	.338	1.00	0.97, 1.03	.946
Median Allowed PMPM (Baseline)	1.00	1.00, 1.00	.453	1.00	1.00, 1.00	.630
Spend in the pre-index month (log)	1.02	0.97, 1.07	.364	0.95	0.85, 1.05	.323
Baseline Use of ACEis	1.11	0.81, 1.53	.510	0.96	0.51, 1.79	.900
Baseline Use of ARBs	1.22	0.88, 1.69	.239	0.98	0.52, 1.87	.954
Baseline Use of Beta Blockers	1.27	0.90, 1.79	.167	1.40	0.67, 2.92	.372
Number of Baseline Therapies	0.84	0.64, 1.10	.202	1.06	0.62, 1.81	.843
Number of Baseline Observation Encounters	0.68	0.40, 1.14	.145	0.55	0.19, 1.63	.283
Cardiologist Visit Before Index	1.10	0.93, 1.30	.259	1.07	0.75, 1.52	.720
Met Reduced EF Criteria	0.96	0.80, 1.15	.624	1.54	0.97, 2.44	.068
Number of All-Cause Admits During Baseline	1.10	0.97, 1.24	.157			
Number of Heart Failure Admits During Baseline				1.22	0.94, 1.58	.134
Number of Observations	2,977			2,977		
Concordance (c) Index	0.682			0.783		

Note: Entries of "-" stand in place of the reference level for categorical variables that have more than two levels. Blank cells indicate that a given variable was not included in the model for the associated outcome. Variables with "[Yes]" at the end of their name indicate two-level categorical variables; their reference level is no evidence of the given procedure or condition. The variable "Dose" (first-listed) is the dose group variable of primary interest in analyses for which pairwise comparisons are reported; this dosage reflects that of the final sacubitril/valsartan fill.
CI = Confidence Interval, CVD = Cardiovascular disease, EF = Ejection fraction, HR = Hazard Ratio, HS = high school, IP = Inpatient, PDC = Proportion of days covered, PMPM = Per-member per-month, SVI = Social Vulnerability Index.

Figure S1. Flow diagram of the selection process for the study sample.

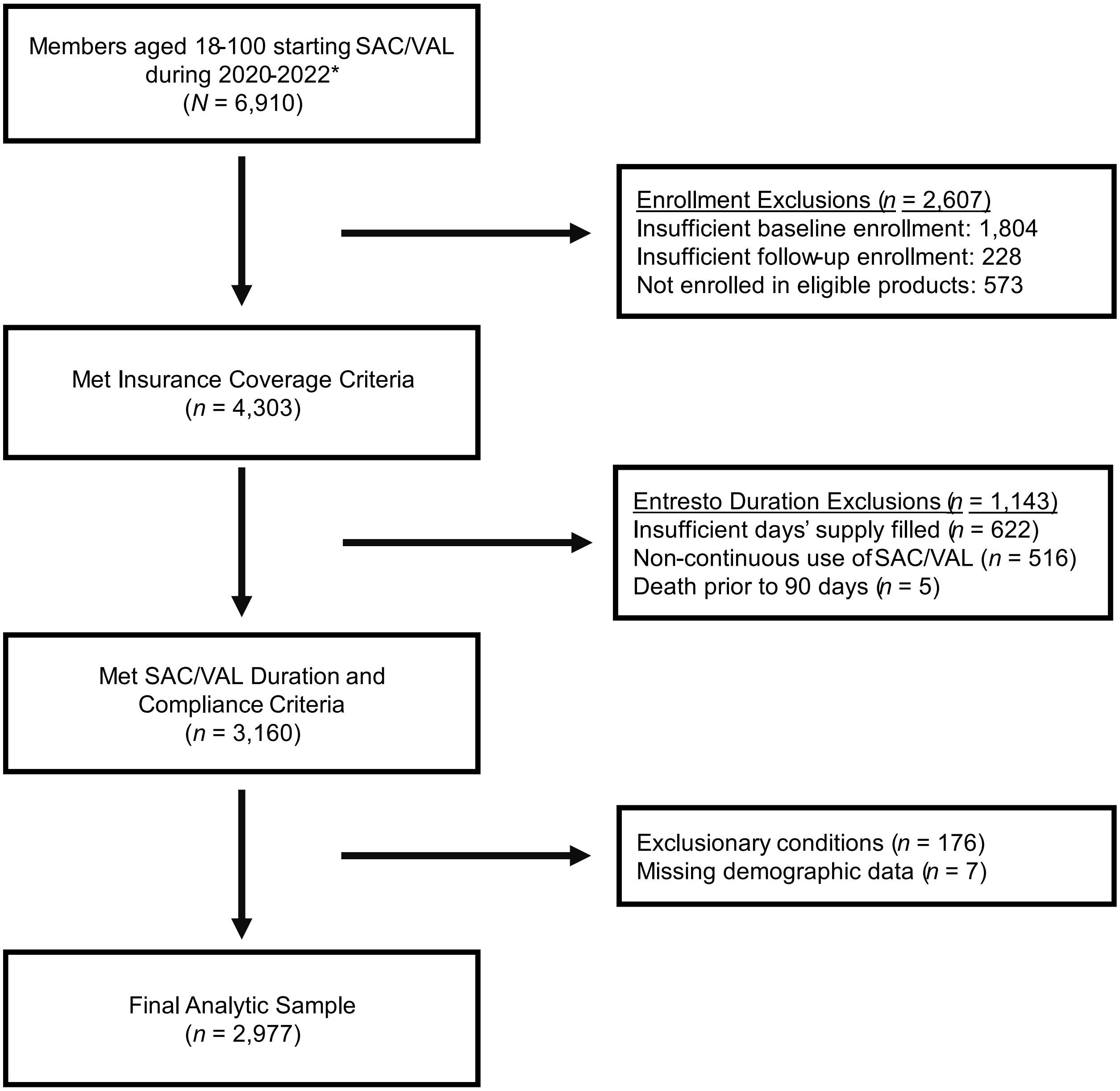

Figure S2. Unadjusted average total allowed spend per member per month (PMPM) as a function of months since starting SAC/VAL with line of best fit and 95% confidence intervals.

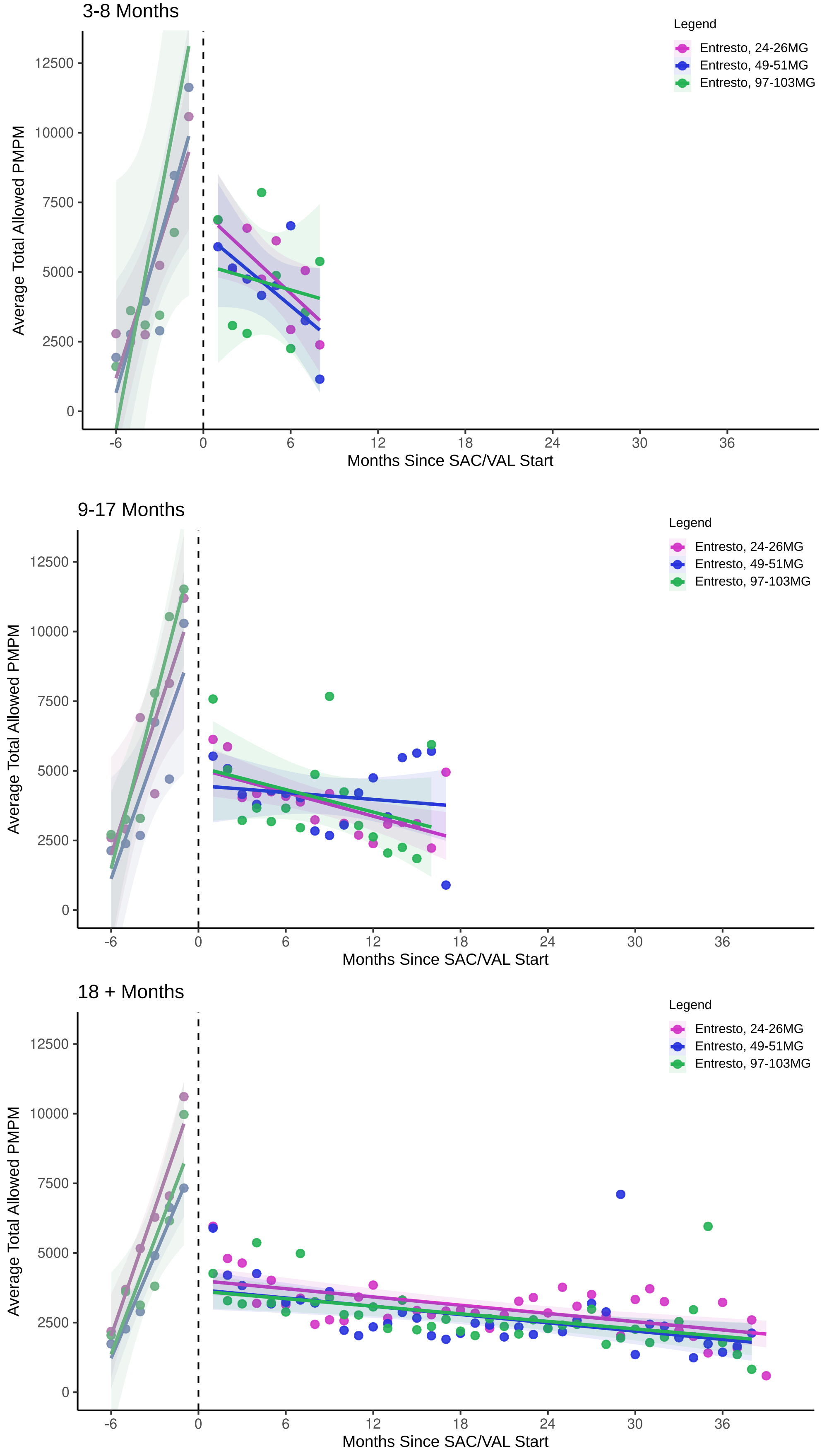

Figure S3. Unadjusted average all-cause inpatient admissions per member per month (PMPM) as a function of months since starting SAC/VAL with line of best fit and 95% confidence intervals.

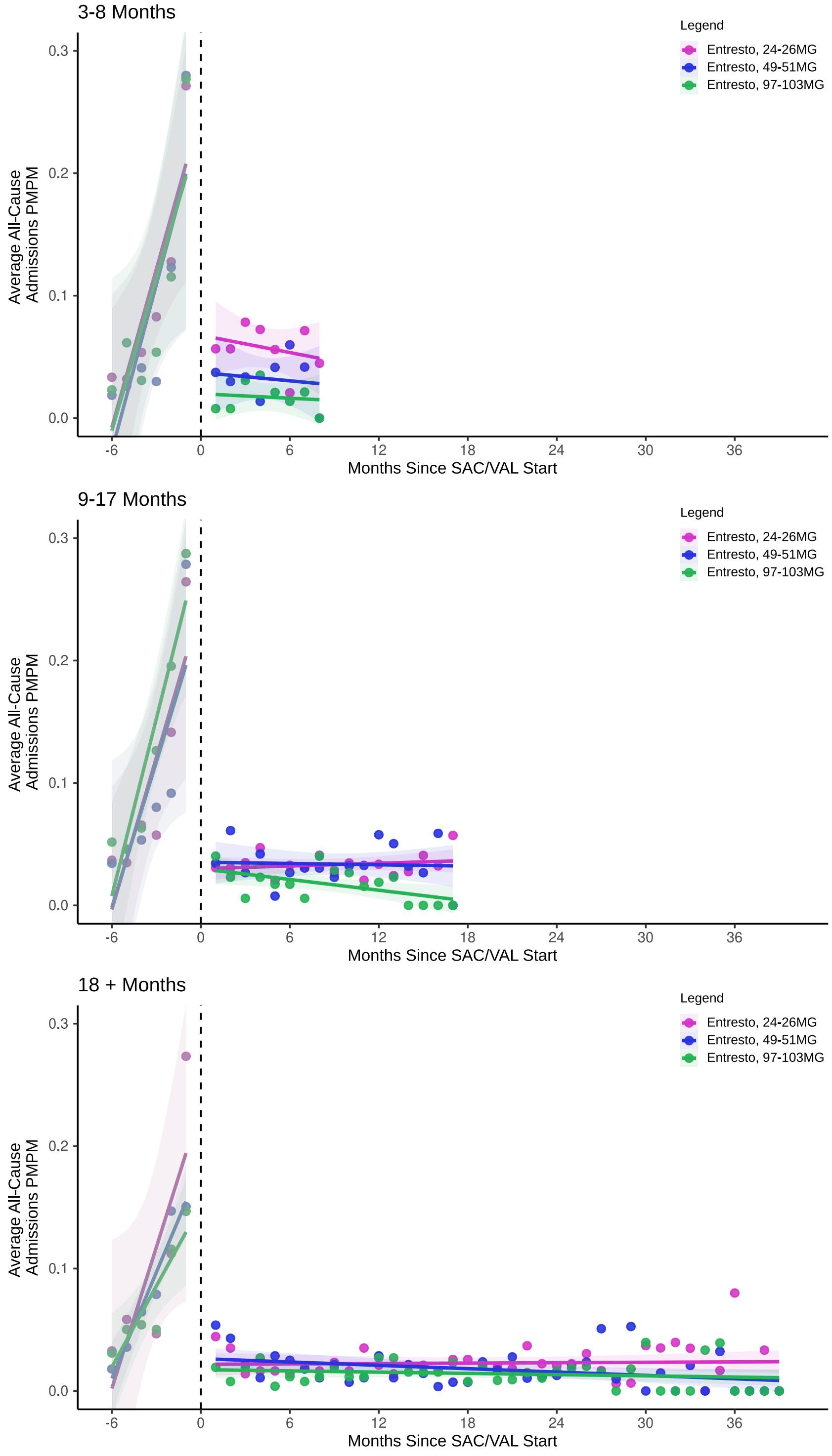

Figure S4. Unadjusted average heart failure admissions per member per month (PMPM) as a function of months since starting SAC/VAL with line of best fit and 95% confidence intervals.

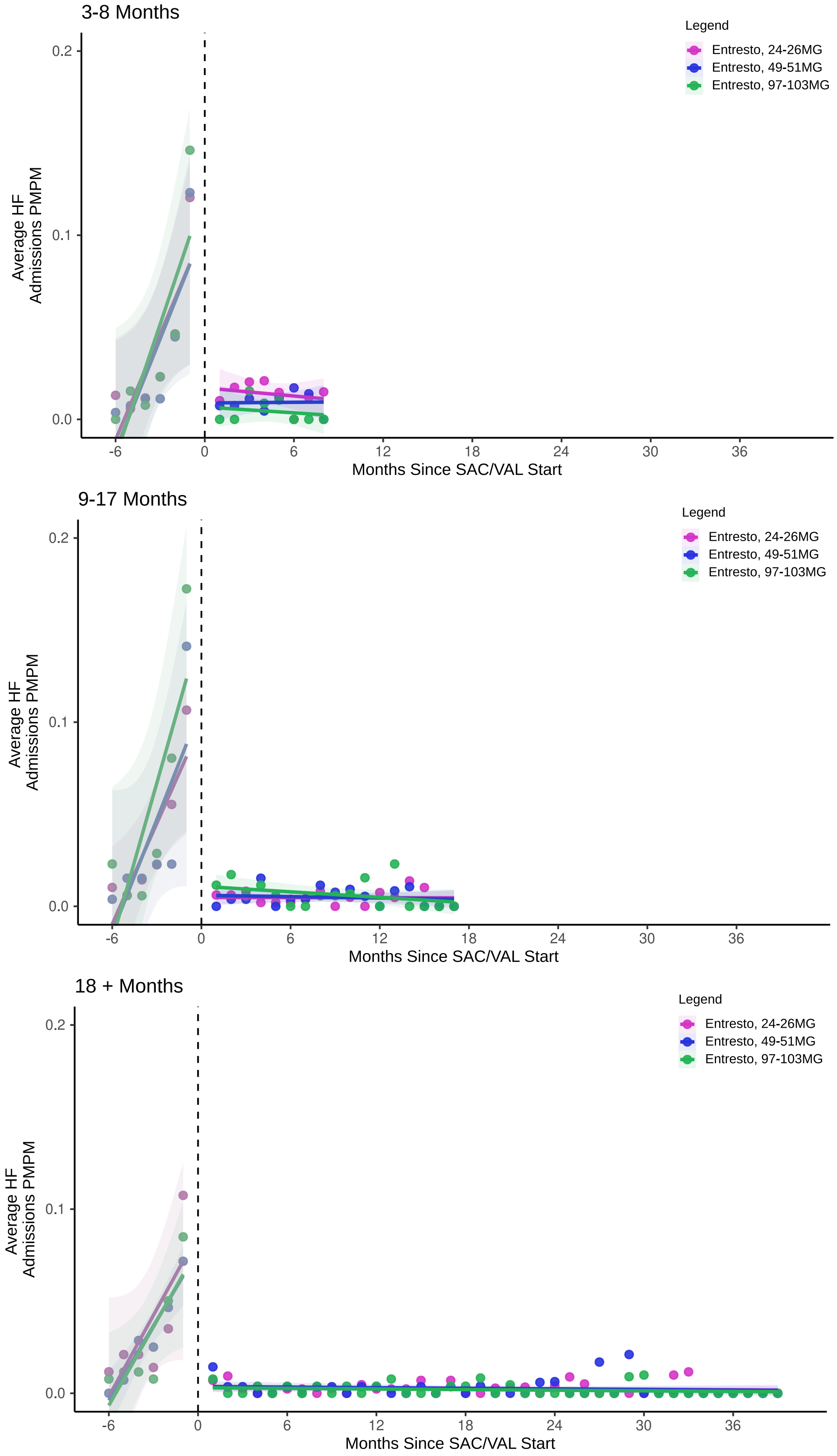

Supplement: S1 File — (PDF) [file pone.0320216.s001.pdf]
