# Supplementary material for: Dosing patterns and dose effects of sacubitril/valsartan: A claims-based retrospective cohort study
Source: PLoS One. 2025 Mar 25;20(3):e0320216. doi: 10.1371/journal.pone.0320216 (PMC11936244; doi:10.1371/journal.pone.0320216)
Supplement: Attachment — Submitted filename: SAC-VAL Responses.docx [file pone.0320216.s002.docx]

Response to Reviewers

Note: Reviewer comments in bold, responses in unbolded, italicized text.

**Reviewer 1**
**This research paper reveals the real world practice of using ARNI in treating patients with heart failure. It showed the importance of up titrating of ARNI to the GDMT recommended dosage to reduce the total cost of treatment of heart failure and hospitalisation due to heart failure. The paper also analysed the likely causes of the failure of this optimisation of the therapy. The failure is most likely caused by physician’s factor (clinical inertia) as no clear explanation to the contraindications to escalate the dosage of ARNI.**

**Though it is a retrospective observational study with a lot of limitations as discussed it still able to send a clear message to physicians to overcome clinical inertia and adhere to clinical guidelines to help patients with heart failure to achieve better clinical outcomes and improve cost effective treatment.**

*We are appreciative of these supportive comments from Reviewer 1.*

**Reviewer 2**

**1.Systolic heart failure is an old definition and current definition is heart failure with reduced ejection fraction. Would it be possible to classify HF by LVEF or is it not possible to obtain this data for the members?**

*Unfortunately, LVEF values are not contained in our claims database, nor do we have access to other information sources that could yield LVEF values for the full sample. Identification and classification of HF is challenging from claims due to the discordance in the ICD-10 CM coding system and definitions used in practice. Most claims algorithms to classify HF are based on the ICD-9 coding system, whereas all the claims in our study were captured after the conversion to ICD-10. The classification scheme we used was based on preliminary findings from Sandhu et al., which is the only report we know of that uses ICD10 codes for HF categorization. They evaluated the concordance between ICD-10 diagnoses from claims and HF types based on test results from EMR among VA patients with HF. They found that certain codes were associated with having EF ≤ 50% (i.e., between 77% to 90% of patients with these codes had mildly reduced, or reduced EF); these are the codes used in our indicator of “systolic HF,” which we have renamed given what this variable is intended to represent (evidence of reduced LVEF). We have added this additional context on the data-related availability and coding to the main-text.*

**2.The author stated that members from the database has identifiable information but the full details of the database is not accessible - Please explain further the implications of this statement, and why this database is nevertheless still valid academically and statistically for this review.**

*The contents of our data base are very similar to those in other claims databases*. *A comparable, publicly documented database would be that for Medicare claims. An example of all the possible information that can be populated for an outpatient encounter can be seen here:* [*https://resdac.org/cms-data/files/op-encounter/data-documentation*](https://resdac.org/cms-data/files/op-encounter/data-documentation)*. The specific fields available varies by claim type, (e.g., outpatient vs. inpatient) but typically involves a similarly large array. While this level of detail is helpful to understand the data source at large, there are many fields that are irrelevant to our study and for most research applications more broadly.*

*Instead of providing the aforementioned type of documentation, we have aimed to make the key information needed for constructing a similar dataset, including our variable definitions, as clear as possible. We feel that this information is more practical and, in many applications, more useful since it will be less particular to the nuances of any given database.*

*There are of course limitations to using claims data for research, though we are not aware of any that are likely unique to, or more pronounced within our database. In response to this reviewer's concerns, we have done the following:*

- *Added information on issues with claims data and those that may be most relevant to our study aims and sample (see pages 18-19, lines 334-351). A new reference has been added in this section, which serves as an example of how coding practices may impact information derived from administrative data.*
- *We have expanded the description of the database, including naming the insurer and the parent organization it belongs to (page 4, lines 73-81).*
- *We now include a description of and findings from a data validation we have performed, as well as a description of the data source used for the validation. In brief, the claims come from a health plan that is part of a blended health organization (i.e., contains both payers and providers), which grants us access to medical records in addition to claims information. We performed a validation in which we compared a select number of member's claims-derived SAC/VAL dosing information to that documented in their medical records. We found perfect agreement between the two sources. This information appears on page 4, lines 78-81 and page 7, lines 158-165.*

*Despite the limitations of our data sources, we feel the careful construction of our data set, the checks performed, and agreement between our findings and others in the literature (i.e., high prevalence of under-dosing, evidence of dose-dependent effects on hospitalizations) supports the validity of our findings.*

*Finally, in terms of data sharing, our study was conducted with member-level data that includes a very large number of specific medical and time-related characteristics. As a point of reference, the level of data deemed permissible for public use and sharing from Medicare claims is extremely limited and abstracted (e.g., diagnosis group to indicate what an outpatient encounter was for; age provided in categories). The level of detail in our analytic dataset is most comparable to Medicare's limited data set (LDS), which is considered identifiable due to the potential to re-identify a member. Links to this information for CMS’s Medicare database are provided below.*

*In lieu of providing the underlying data, we have provided a significant amount of aggregate data for the sample (overall and stratified by dose group), for both the main outcomes and member characteristics, as well as extensive details for all modeling analyses conducted. Should a reader be interested in additional aggregate information not currently present, we are happy to assist; but for further information on the limitations of sharing we defer to our local IRB as stated in the data availability.*

*Medicare data set types and characteristics*

<https://resdac.org/articles/differences-between-rif-lds-and-puf-data-files>

*LDS storage standards (see #5)*

<https://www.cms.gov/files/document/lds-dua-terms-and-conditions.pdf>
